# Refined Composite Multiscale Fuzzy Dispersion Entropy and Its Applications to Bearing Fault Diagnosis

**DOI:** 10.3390/e25111494

**Published:** 2023-10-29

**Authors:** Mostafa Rostaghi, Mohammad Mahdi Khatibi, Mohammad Reza Ashory, Hamed Azami

**Affiliations:** 1 Modal Analysis (MA) Research Laboratory, Faculty of Mechanical Engineering, Semnan University, Semnan 35131-19111, Iran; rostaghi@semnan.ac.ir (M.R.); mashoori@semnan.ac.ir (M.R.A.); 2 Centre for Addiction and Mental Health, University of Toronto, Toronto, ON M6J 1H1, Canada; hmdazami@gmail.com

**Keywords:** fuzzy dispersion entropy, refined composite multiscale, fault feature extraction, bearing fault diagnosis

## Abstract

Rotary machines often exhibit nonlinear behavior due to factors such as nonlinear stiffness, damping, friction, coupling effects, and defects. Consequently, their vibration signals display nonlinear characteristics. Entropy techniques prove to be effective in detecting these nonlinear dynamic characteristics. Recently, an approach called fuzzy dispersion entropy (DE–FDE) was introduced to quantify the uncertainty of time series. FDE, rooted in dispersion patterns and fuzzy set theory, addresses the sensitivity of DE to its parameters. However, FDE does not adequately account for the presence of multiple time scales inherent in signals. To address this limitation, the concept of multiscale fuzzy dispersion entropy (MFDE) was developed to capture the dynamical variability of time series across various scales of complexity. Compared to multiscale DE (MDE), MFDE exhibits reduced sensitivity to noise and higher stability. In order to enhance the stability of MFDE, we propose a refined composite MFDE (RCMFDE). In comparison with MFDE, MDE, and RCMDE, RCMFDE’s performance is assessed using synthetic signals and three real bearing datasets. The results consistently demonstrate the superiority of RCMFDE in detecting various patterns within synthetic and real bearing fault data. Importantly, classifiers built upon RCMFDE achieve notably high accuracy values for bearing fault diagnosis applications, outperforming classifiers based on refined composite multiscale dispersion and sample entropy methods.

## 1. Introduction

Rotating machines, such as gas turbines, industrial fans, aero-engines, gearboxes, and wind turbines, are widely used in different industrial and mechanical applications [1]. Rolling bearings are one of the most crucial and extensively used components in most rotating machines [2]. Because of improper initial assembly, low accuracy in manufacturing, and repetitively applied stress, bearing faults are unavoidable in long-term operations [3]. If bearings are not diagnosed and replaced promptly, it can cause disruptions in the operation of machines. For instance, bearing failures are accountable for around 40–50% of all failures that occur in electrical motors [4]. Thus, early fault detection using vibration data decreases maintenance costs and increases reliability [5,6].

Vibration signals involve the information on the dynamic features of machines and structures. Hence, their analysis is one of the conventional fault detection methods in rotating machines. Vibration signals generally represent nonlinear behavior due to effects associated with coupling, interactions, friction, damping, and nonlinear stiffness [7,8]. Therefore, the capabilities of linear feature extraction techniques have been limited in fault diagnosis [9], and researchers have focused on detecting nonlinear dynamical characteristics to improve fault diagnosis capabilities [10,11].

Due to various faults in ball bearings, including inner race faults, outer race faults, and rolling element faults, impacts with frequencies associated with the faults are generated. These impacts lead to the resonant excitation of the bearing. As a result, in each fault signal, the impacts are modulated by the much higher resonant frequencies of the bearing [12]. As a result, each fault creates different changes in signal complexity at different time scales. Hence, measuring signal complexity by calculating entropy over different scales (multiscale algorithms) is extensively applied to bearing fault diagnoses [13,14,15].

Entropy, as a measure of the irregularity and unpredictability of signal, is a powerful concept employed for evaluating the nonlinear features of a signal [16]. Sample entropy (SE), fuzzy entropy (FE), permutation entropy (PE) and fuzzy entropy (FE) are common entropy methods. Their advantages, disadvantages and some references of biomedical and mechanical applications are presented in Table 1.

Nevertheless, DE is sensitive to its parameters, particularly the number of classes and embedding dimension [19]. To overcome these limitations, we have recently developed fuzzy DE (FDE) based on fuzzy membership functions for signal quantization and DE [19]. However, similarly to SE, FE, PE, and DE, FDE is unable to consider the multiple time scales that are present in data.

In order to overcome this limitation, Costa et al. [36] proposed multiscale SE (MSE) as a method for estimating the complexity of univariate data using the coarse-graining (CG) process. However, MSE inherits the SE limitations. Similarly, multiscale PE [37] and multiscale FE [38] have the shortcoming of PE and FE, respectively.

Apart from that, the CG procedure causes the signal length to be shorter as the scale factor increases. As a result, the higher the scale factor, the lower the accuracy of entropy [39]. To address this problem, Wu et al. [40] proposed composite MSE (CMSE) to improve the accuracy of MSE. Afterwards, proposing refined composite MSE (RCMSE), Wu et al. [41] decreased the probability of the undefined values of SE in the multiscale algorithm in addition to improving the accuracy of MSE. Multiscale algorithm refinement is also conducted in other studies, including refined composite multiscale fuzzy entropy (RCMFE) by Azami et al. [42], refined composite multiscale permutation entropy (RCMPE) by Humeau-Heurtier et al. [43], and refined composite multiscale dispersion entropy (RCMDE) by Azami et al. [33].

Wang et al. [44] used MDE to extract the features of bearing vibration signals, while Congzhi et al. [45], Zhang et al. [46], and Luo et al. [47] employed RCMDE for this purpose. Also, different techniques are applied with RCMDE, including fast ensemble empirical mode decomposition [48], adaptive sparest narrow-band decomposition [49], improved empirical wavelet transform [50,51], variational mode decomposition (VMD) [52], and improved VMD [14].

Nevertheless, all above-mentioned multiscale algorithms have the shortcomings of their corresponding entropy algorithms. To address these shortcomings, based on the advantages of FDE over existing entropy algorithms, multiscale fuzzy dispersion entropy (MFDE) and refined composite MFDE (RCMFDE) are developed in this article. The ability of these methods is evaluated by various synthetic and real datasets.

This paper is structured as follows: In Section 2.1 and Section 2.2, the descriptions of MFDE and RCMFDE are provided, respectively. Section 3 briefly describes the synthetic and real datasets used in this study. In Section 4.1, Section 4.2 and Section 4.3, the ability of RCMFDE to calculate complexities associated with white noise, logistic map series, and chirp signal is compared to MDE, RCMDE, and MFDE. In Section 4.4 and Section 4.5, the sensitivity to the signal length and the computation time of RCMFDE are investigated. Section 4.6 and Section 4.7 present the proposed method’s capability to distinguish between faulty and healthy bearings for simulated signals and the effect of noise on its performance. Section 4.8 evaluates the performance of the proposed method in fault diagnosis using three different datasets. Finally, Section 5 provides a conclusion.

## 2. Methods

In this paper, FDE is extended to RCMFDE and MFDE. This method is explained as follows.

### 2.1. Multiscale Fuzzy Dispersion Entropy

#### 2.1.1. Coarse-Graining

The assessment of complexity in univariate signals is often accomplished via the utilization of a multiscale entropy framework, which encompasses two fundamental steps: the process of coarse graining to encompass multiple temporal scales and the evaluation of irregularity for each of these scales using entropy estimators.

$x^{\left(\tau \right)}=\left\{x_{1}^{\left(\tau \right)},x_{2}^{\left(\tau \right)},\ldots \right\}$ at scale τ of series $u=\left\{u_{1},u_{2},\ldots ,u_{L}\right\}$ of length *L* is defined as follows:

(1) $$x_{j}^{\left(\tau \right)}=\frac{1}{\tau }\sum_{b=\tau (j-1)+1}^{\tau j}u_{b},1\leq j\leq \left\lfloor \frac{L}{\tau }\right\rfloor$$

#### 2.1.2. Multiscale Fuzzy Dispersion Entropy Calculation

MFDE calculates FDE over some consecutive temporal scales. The FDE of each coarse graining signal $x^{\left(\tau \right)}$ is calculated. What is of note is that the average and standard deviation (SD) of the original signal remain unchanged for all scale factors, in agreement with keeping parameter *r* constant when calculating MSE [36].

#### 2.1.3. Fuzzy Dispersion Entropy

Fuzzy dispersion entropy (FDE) for time series $x=\{x_{1},x_{2},\ldots ,x_{N}\}$ of length N can be calculated using six steps [19]:

Step (1): First, time series **x** is normalized between 0 and 1 to obtain $y=\{y_{1},y_{2},\ldots ,y_{N}\}$. Different linear and non-linear methods can be employed for this normalization [16,18]. However, the utilization of a linear mapping technique may result in an issue where the majority of *x_i_* values are assigned to only a few classes, especially when the maximum or minimum values deviate significantly from the signal’s mean/median values [16]. Consequently, DE with linear mapping exhibits poor performance in characterizing signals [16,18]. Many natural processes follow a pattern that starts slowly and accelerates, ultimately approaching a climax over time, similarly to a sigmoid function [7,53,54]. In cases where a detailed description is unavailable, a sigmoid function is commonly used [53,55]. Various sigmoid functions are available [18]. In accordance with the original formulation of DE [16], the normal cumulative distribution function, as a widely recognized sigmoid, was used. Series y is obtained from the normal cumulative distribution function of series x according to Equation (2):

(2) $$y_{i}=\frac{1}{\sigma \sqrt{2\pi }}\int_{-\infty }^{x_{i}}e^{\frac{{\left(t-\gamma \right)}^{2}}{2\sigma^{2}}}dt$$

where σ and γ are the SD and average of time series x.

Step (2): In this step, time series y is mapped onto classified time series $z^{c}$ [16]. Each $y_{i}$ is multiplied by c and summed with 0.5.

(3) $$z_{i}^{c}=c\cdot y_{i}+0.5$$

where $z_{i}^{c}$ is the *i*^th^ member of series $z^{c}$, and c∈ℕ is the class parameter indicating the number of all classes that can belong to $z_{i}^{c}$ [16].

Step (3): In DE, $z_{i}^{c}$ belongs to the *k*^th^ class if it is closer to integer *k* [16]. Since ambiguity in allocating the members of series $z^{c}$ occurs in the boundaries of two classes, a fuzzy membership function $M_{k}$ is defined for each class, and $\mu_{M_{k}}(z_{i}^{c})$ represents the degree of membership of $z_{i}^{c}$ with respect to the *k*^th^ class. Every $z_{i}^{c}$ is assigned to one or two classes (using integer indexes where k=1,…,c).

For designing a fuzzy membership function related to each class, the following conditions must be met:

1. There is no boundary at the starting points of class 1 and end points of class c with other classes. Therefore, if $z_{i}^{c}$ is lower than 1 and higher than *c*, its degree of membership to classes 1 and *c* is equal to 1.
2. The sum of the membership values of $z_{i}^{c}$ in different classes must be equal to 1.
  (4) $$\sum_{k=1}^{c}\mu_{M_{k}}\left(z_{i}^{c}\right)=1$$
3. For a series of random numbers, the fuzzy membership functions possess equal relative cardinality.
4. The overlap of the fuzzy membership function of each class with that of the adjoining classes can be 1 at most.

As mentioned above, different membership functions can be applied. This study employs triangular membership functions for classes k=2,…,c−1 and trapezoidal membership functions for classes 1 and c. The applied fuzzy functions are expressed as follows:

(5) $$\mu_{M_{1}}\left(\alpha \right)=\{\begin{array}{c} 0\;\;\;\;\;\;\;\;\;\;\;\;\;\;\;\;\;\;\;\alpha >2\\ 2-\alpha \;\;\;\;\;\;\;\;\;1\leq \alpha \leq 2\\ 1\;\;\;\;\;\;\;\;\;\;\;\;\;\;\;\;\;\;\;\alpha <1 \end{array}$$

(6) $$\mu_{M_{k}}\left(\alpha \right)=\{\begin{array}{c} 0\;\;\;\;\;\;\;\;\;\;\;\;\;\;\;\;\;\;\;\;\;\;\alpha <k+1\\ k+1-\alpha \;\;\;\;\;\;\;\;\;\;\;\;\;k\leq \alpha \leq k+1\\ \alpha -k+1\;\;\;\;\;\;\;\;\;\;\;\;\;k-1\leq \alpha \leq k\\ 0\;\;\;\;\;\;\;\;\;\;\;\;\;\;\;\;\;\;\;\;\;\;\;\alpha <k-1 \end{array}\;\;\;\;\;\;\;\;\;\;\;\;\;k=2,\ldots ,c-1$$

(7) $$\mu_{M_{c}}\left(\alpha \right)=\{\begin{array}{c} 1\;\;\;\;\;\;\;\;\;\;\;\;\;\;\;\;\;\;\;\;\;\;\;\alpha >c\\ \alpha -c+1\;\;\;\;\;\;\;c-1\leq \alpha \leq c\\ 0\;\;\;\;\;\;\;\;\;\;\;\;\;\;\;\;\;\;\;\;\;\;\alpha <c-1 \end{array}$$

Step (4): Time series $z_{j}^{m,c}$ with time delay d and embedding dimension m is constructed according to Equation (8):

(8) $$z_{j}^{m,c}=\left\{z_{j}^{c},z_{j+(1)d}^{c},\ldots ,z_{j+(m-1)d}^{c}\right\},j=1,2,\ldots ,N-(m+1)d$$

Step (5): Dispersion patterns $\pi_{v_{0}v_{1}\ldots v_{m-1}}$ in the context of dispersion entropy refer to the distribution of data points within each embedded time series of length *m*. These embedded time series are generated by embedding the digitized versions of the original time series data into discrete classes [16,18].

**Figure 1:**
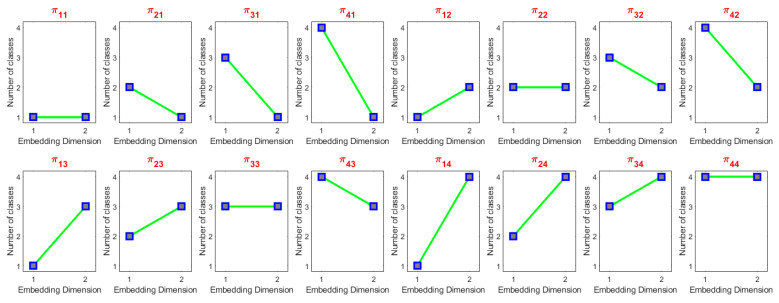
All possible dispersion patterns for *m* = 2 and *c* = 4.

For each time series embedded into the dimensions of *m* and a given number of classes *c*, there exists a potential for the occurrence of $c^{m}$ dispersion patterns. Figure 1 illustrates all potential dispersion patterns for *m* = 2 and *c* = 4.

Each vector $z_{j}^{m,c}$ is mapped onto different dispersion patterns $\pi_{v_{0}v_{1}\ldots v_{m-1}}$ based on its membership values so that the following is the case:

$z_{j}^{c}\;\mathrm{is}\;\mathrm{class}\;v_{0}\;\mathrm{and}\;z_{j+(1)d}^{c}\;\mathrm{is}\;\mathrm{class}\;v_{1},\ldots ,\;\mathrm{and}\;z_{j+(m-1)d}^{c}\;\mathrm{is}\;\mathrm{class}\;v_{m-1}\;,\;\mathrm{if}\;\mathrm{and}\;\mathrm{only}\;\mathrm{if}\;z_{j}^{m,c}\;\mathrm{is}\;\pi_{v_{0}v_{1}\ldots v_{m-1}}.$ When multiple states need to be true simultaneously (i.e., AND operator), *t-norm* is applied. The algebraic product operator is a *t-norm*, which is applied in the above fuzzy expression [19]:

(9) $$\mu_{\pi_{v_{o}v_{1}\ldots v_{m-1}}}\left(z_{j}^{m,c}\right)=\prod_{i=0}^{m-1}\mu_{M_{v_{i}}}\left(z_{j+i\cdot d}^{c}\right)$$

According to Equation (9), the degree of membership of $z_{j}^{m,c}$ with respect to the pattern $\pi_{v_{0}v_{1}\ldots v_{m-1}}$ is equal to the product of the degree of membership of each $z_{j+i\cdot d}^{c}$ with respect to class $v_{i}$, where i=0,1,…,m−1 and j=1,2,…,N−(m−1)d.

Step (6): For each $c^{m}$ of dispersion pattern $\pi_{v_{0}v_{1}\ldots v_{m-1}}$, the probability of presence in the time series is calculated. For this purpose, the sum of the membership degrees of dispersion patterns $\pi_{v_{0}v_{1}\ldots v_{m-1}}$, attributed to all series $z_{j}^{m,c}$, must be divided by the total number of embedded signals with embedding dimension *m*:

(10) $$p(\pi_{v_{0}v_{1}\ldots v_{m-1}})=\frac{\sum_{j=1}^{N-(m-1)d}\mu_{\pi_{v_{0}v_{1}\ldots v_{m-1}}}\left(z_{j}^{m,c}\right)}{N-(m-1)d}$$

Step (7): Finally, based on Shannon entropy, FDE is calculated as follows:

(11) $$FDE(x,m,c,d)=-\sum_{\pi =1}^{c^{m}}P(\pi_{v_{0}v_{1}\ldots v_{m-1}})\cdot \ln P(\pi_{v_{0}v_{1}\ldots v_{m-1}})$$

### 2.2. Refined Composite Multiscale Fuzzy Dispersion Entropy

In calculating RCMFDE, for scale factor τ, different τ time series are created, corresponding to different starting points of the CG process. The *k*^th^ coarse-grained time series $x_{k}^{\left(\tau \right)}=\left\{x_{k,1}^{\left(\tau \right)},x_{k,2}^{\left(\tau \right)},\ldots \right\}$ of the original time series $u=\left\{u_{1},u_{2},\ldots ,u_{L}\right\}$ is obtained as follows:

(12) $$x_{k,j}^{\left(\tau \right)}=\frac{1}{\tau }\sum_{b=k+\tau (i-1)}^{k+\tau j-1}u_{b},1\leq j\leq L,1\leq k\leq \tau$$

The relative frequency of the fuzzy dispersion patterns of each of the τ time series is calculated.

The Shannon entropy of the average relative frequencies of fuzzy dispersion patterns for the τ time series created by different beginning points in the CG process is equal to RCMFDE. Therefore, RCMFDE for each scale factor is defined as follows:

(13) $$\mathrm{RCMFDE}(x,m,c,d,\tau )=-\sum \bar{p}(\pi_{v_{0}v_{1}\ldots v_{m-1}}).\ln(\bar{p}(\pi_{v_{0}v_{1}\ldots v_{m-1}}))$$

In this equation, $\bar{p}\left(\pi_{v_{0}v_{1}\ldots v_{m-1}}\right)=\frac{1}{\tau }\sum_{1}^{\tau }p_{k}^{\left(\tau \right)}\left(\pi_{v_{0}v_{1}\ldots v_{m-1}}\right),$ where $p_{k}^{\left(\tau \right)}\left(\pi_{v_{0}v_{1}\ldots v_{m-1}}\right)$ is the relative frequency of fuzzy dispersion pattern $\pi_{v_{0}v_{1}\ldots v_{m-1}}$ in time series $x_{k}^{\left(\tau \right)}$.

The number of possible dispersion patterns is recommended to be lower than the signal length $\left(c^{m}<L\right)$. For MFDE, the CG process decreases the signal length to $\left\lfloor \frac{L}{\tau_{\max}}\right\rfloor$. Thus, for MFDE, it is suggested that $c^{m}<\left\lfloor \frac{L}{\tau_{\max}}\right\rfloor$. In RCMFDE, the τ coarse-grained time series of length $\left\lfloor \frac{L}{\tau_{\max}}\right\rfloor$ are taken into account. Therefore, the number of all samples calculated in RCMFDE is $\tau \times \left\lfloor \frac{L}{\tau_{\max}}\right\rfloor \approx L,$ and RCMFDE with the same length of $c^{m}<L$ gives reliable results. This particular feature is a matter of importance in short signals.

This study utilizes parameters *m* = 2, *c* = 3, and *d* = 1 to compute MDE, RCMDE, MFDE, and RCMFDE.

## 3. Evaluation Signals

To evaluate the effectiveness of RCMFDE to characterize different univariate time series and bearing fault diagnosis, we employ the following synthetic and bearing datasets.

### 3.1. Synthetic Signals

#### 3.1.1. White Gaussian Noise and 1f Noise

White Gaussian noise (WGN) and 1f noise (pink noise) have been widely used for evaluating multiscale entropy techniques since WGN is less complex but more irregular than pink noise [33,56,57,58].

#### 3.1.2. Logistic Map

The logistic map is a simple mathematical model that plays a key role in chaos theory since it illustrates the emergence of chaotic behavior from a relatively simple nonlinear equation. It is often used as a prototype example to understand the dynamics of chaotic systems [59]. The logistic map time series $x=\left\{x_{1},x_{2},\ldots ,x_{n}\right\}$ is defined as follows [60,61]:

(14) $$x_{i+1}=r\cdot x_{i}\cdot (1-x_{i}),x_{1}=0.1,\;i=1,2,3,\ldots$$

where $x_{i}$ shows the logistic map at time step *i*, and it symbolizes the population at year *i*. As a result, $x_{0}$ signifies the initial population at time step 0, specifically set as $x_{0}=0.1$. The parameter *r* functions as a control factor, representing a positive combined rate that encompasses both reproduction and starvation effects [62]. The first $10^{5}$ iterations of Equation (14) are ignored due to the transient behavior of the solution [63]. Chaotic behaviors occur for 3.57≤r≤4 [61]. The logistic map was used to evaluate the performance of MFDE and RCMFDE in estimating the complexity of data.

#### 3.1.3. Chirp Signal and Amplitude-Modulated Chirp Signal

To investigate the relationship of the proposed methods with variations in time and frequency domains, two types of signals were synthesized. For the first type of signal, a constant-amplitude chirp signal was selected, with its frequency logarithmically varying within the range of 2 to 15 Hz. For the second type, the same signal as the first type was modulated with a pure sinusoidal wave. These two signals were generated with a sampling frequency of 100 Hz and a duration of 40 s.

#### 3.1.4. Faulty Bearing Simulation

A local fault in a bearing produces a periodic impact signal that leads to the resonant excitation of the bearing; therefore, it is modulated by the significantly higher resonant frequencies of the bearing [12]. The simulated vibration signal for a bearing of a rotating machine with outer ring damage is defined as follows [8]:

(15) $$x(t)=x_{\mathrm{series}\;\mathrm{of}\;\mathrm{impulses}}+x_{\mathrm{harmonic}\;\mathrm{component}}+n(t)$$

where $x_{seriesofimpulses}$ and $x_{harmoniccomponent}$ are the series of impulses and the harmonic component, respectively. Also, n(t) represents noise. Based on previous studies [8,64,65], $x_{seriesofimpulses}$ is modeled by Equation (14) [8].

(16) $$x_{\mathrm{series}\;\mathrm{of}\;\mathrm{impulses}}=\sum_{k=1}^{m}\sum_{n=1}^{n^{\prime }}Ae^{-2\xi \pi f_{n}(t-\frac{k}{f_{f}}-\sum_{i=1}^{k}\tau_{k})}\sin(2\pi f_{n}\sqrt{1-\xi^{2}}(t-\frac{k}{f_{f}}-\sum_{i=1}^{k}\tau_{k}))$$

$\tau_{k}$ is a representation of a small and random fluctuation in the time interval between two successive impulses. The frequency of the impulse train signal is considered equal to $f_{f}$ without taking into account the impact of accidental slipping by the balls and taking into account a constant period. Nevertheless, considering the ball, the slipping effect changes the period randomly to $\frac{k}{f_{f}}-\tau_{k}$ [8]. For each *k*, $\tau_{k}$ is assumed to be a random number from a normal distribution of zero average and SD $\sigma_{\tau }=0.005\times \frac{1}{f_{f}}$ [8].

Equation (17) defines the harmonic component with two sine functions [12,66]:

(17) $$x_{\mathrm{harmonic}\;\mathrm{component}}=\sum_{m=1}^{2}B_{m}\sin(2\pi mf_{r}t)$$

This paper assumes the fault characteristic frequency and damping coefficient as $f_{f}=100\mathrm{Hz}$ and ξ=0.03, respectively. Also, $f_{1}=2k\mathrm{Hz}$ and $f_{2}=3.5k\mathrm{Hz}$ are the resonant frequencies of the bearing. The impulse amplitude magnitude factors are $A_{1}=0.4$ and $A_{2}=0.5$, which specifies the damage intensity. The first and second harmonic amplitudes of the rotor are $B_{1}=0.2$ and $B_{2}=0.12$, respectively.

### 3.2. Bearing Datasets

#### 3.2.1. Paderborn University Dataset

Ball bearing data were provided by the KAT datacenter in Paderborn University [67,68]. The experimental setup includes an electric motor, a torque meter, a flywheel, a bearing test module, and motor load. Bearings with different fault conditions are mounted on the test module to produce experimental data.

Datasets used in this paper involve four different bearing fault conditions: (1) healthy condition (H), (2) sharp trench on the outer ring (STO) by electrical discharge machining, (3) drilling on the outer ring (DO), and (4) artificial pitting on the outer ring (PO) by electric engraver. The used datasets are listed in Table 2. The vibration signals of rolling bearings in different operational states were gathered using a sampling frequency of 64,000 Hz, as demonstrated in Table 3.

#### 3.2.2. PHMAP 2021 Data Challenge Dataset

A subset of the PHMAP 2021 data challenge dataset [54] was utilized. The equipment under investigation comprises an oil injection screw compressor featuring a 15 kW motor operating at 3600 rpm and a screw axis rotating at 7200 rpm. The data collected for this study were acquired using an accelerometer installed on the motor, with a sampling frequency of 10,544 Hz.

Datasets used in this paper involve three different fault conditions: (1) high V-belt looseness, (2) defective bearing, and (3) fault-free state.

#### 3.2.3. Case Western Reserve University Dataset

The Case Western Reserve University (CWRU) dataset [69] was also employed to evaluate the proposed method’s performance in the discrimination of bearing faults. The dataset prepared by the bearing data center of CWRU is a standard reference in the field bearing fault diagnosis [70]. The experimental setup used in data acquisition includes a three-phase electric motor of 2 hp power, a dynamometer, and a self-aligning coupling.

In this study, 6205-2RS JEM SKF ball bearings were used. Single-point faults with different diameters were created on bearings via electrical discharge machining. Bearing vibration signals were gathered from an accelerometer mounted on the casing at the drive end of the motor.

## 4. Results and Discussion

### 4.1. Analysis of White Gaussian Noise and 1f Noise

**Figure 2:**
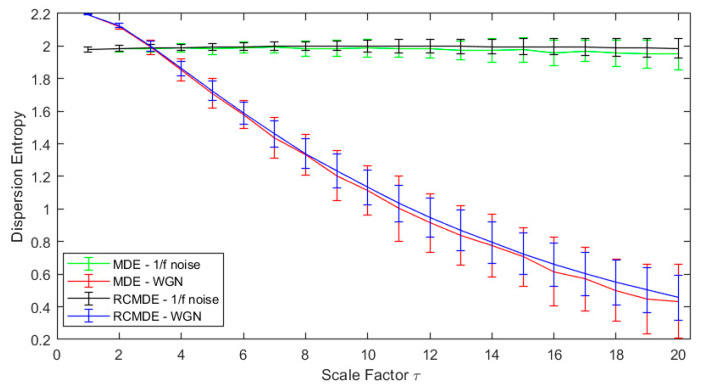
RCMDE and MDE at 20 scales for white and pink noise.

**Figure 3:**
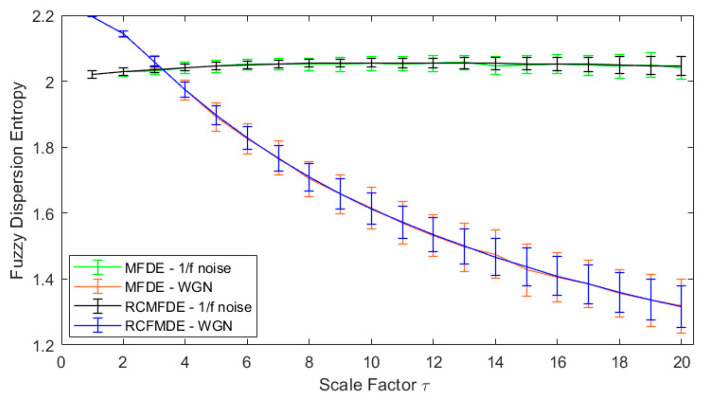
RCMFDE and MFDE at 20 scales for white and pink noises.

One hundred independent white Gaussian and one hundred independent pink noise series of 1000 data point length were created. MFDE, MDE, RCMDE, and RCMFDE were then applied to these signals for scale factors 1–20 (Figure 2 and Figure 3).

In all the examined methods, the entropy of the time series of coarse-grained pink noise is kept almost constant, while it is reduced uniformly by increasing the scale for the WGN. Consequently, at low scale factors (τ≤3), the entropy of white noise is higher than the pink one. At high scale factors (τ>3), the entropy of pink noise is higher than that for the white noise. These results confirm the fact that the complexity of pink noises is higher than WGN while the uncertainty of WGN is more than that for the pink noise [56,57,71].

**Figure 4:**
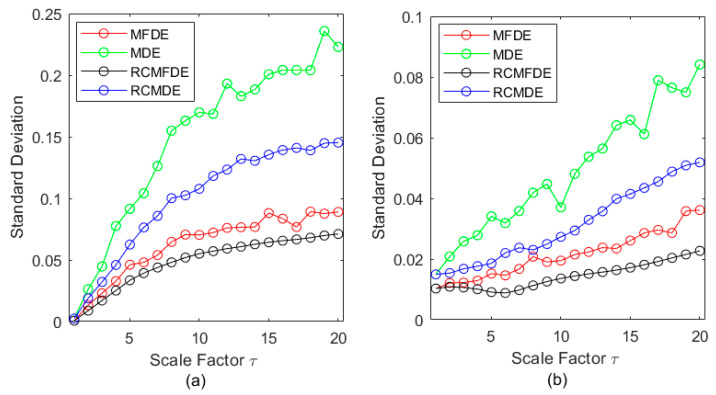
SD of MDE, MFDE, RCMDE, and RCMFDE for one-hundred independent (**a**) white noise and (**b**) pink noise time series.

To assess the stability of the MDE, MFDE, RCMDE, and RCMFDE results, we calculated the SD of their results at each scale factor (Figure 4). The SD values suggest that MFDE and RCMDE, respectively, are more stable than MDE and RCMDE, and the lowest SD was obtained based on RCMFDE.

### 4.2. Analysis of Logistic Map

**Figure 5:**
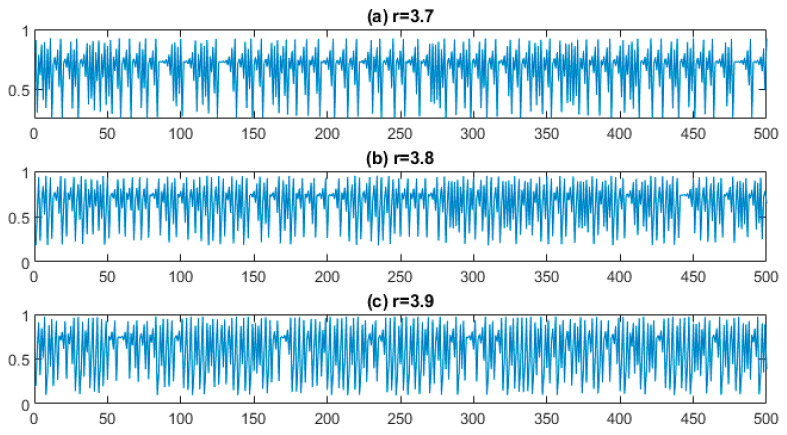
The waveforms resulting from the logistic map change: r = 3.7 (**a**), 3.8 (**b**), and 3.9 (**c**).

Three logistic map series $x=\left\{x_{10^{5}+1},x_{10^{5}+2},\ldots ,x_{10^{5}+500}\right\}$ of length 500 samples with parameters of r={3.7, 3.8, 3.9} based on Equation (14) are created, as shown in Figure 5. Theoretically, the complexity of four signals increases by increasing *r*.

**Figure 6:**
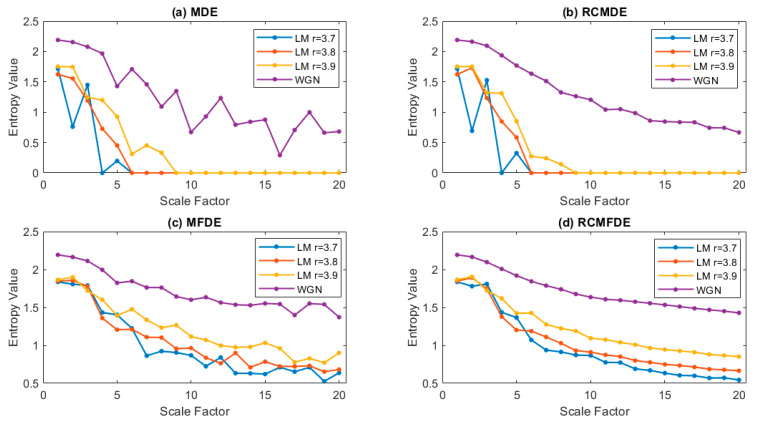
The complexity of logistic map signals with different *r* values and WGN. (**a**) MDE, (**b**) RCMDE, (**c**) MFDE, and (**d**) RCMFDE.

RCMFDE, MFDE, MDE, and MFDE are calculated for these three series and one WGN for scales 1–20 (Figure 6). The results show that RCMFDE is the only method that, after scale 5 (6 to 20), exhibits curves that conform with the arrangement of complexity among different *r* values. The results suggest that RCMFDE is the most appropriate measure of complexity in comparison with MDE, RCMDE, and MFDE.

### 4.3. Analysis of Chirp signals and Amplitude-Modulated Chirp Signal

**Figure 7:**
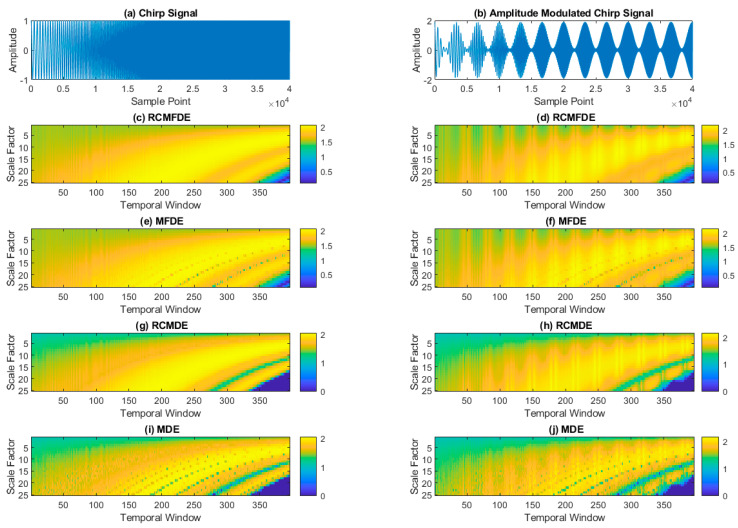
The results of (**c**) RCMFDE, (**e**) MFDE, (**g**) RCMDE, and (**i**) MDE on (**a**) chirp signals in comparison to the results of (**d**) RCMFDE, (**f**) MFDE, (**h**) RCMDE, and (**j**) MDE on (**b**) amplitude-modulated chirp signals.

One chirp signal and one modulated chirp signal, as described in the section, are used to investigate the impact of domain and frequency variations of sinusoidal signals. These two signals are depicted in Figure 7a,b. A moving window of length of 500 samples slid over the signals with an 80% overlap between windows. For each isolated signal, the values of RCMFDE, MFDE, RCMDE, and MDE were computed.

As depicted in Figure 7c,e,g,i, all methods adeptly capture frequency variations. In the initial segments of the signals where the frequency is lower, smaller scale values exhibit higher entropy. Conversely, as frequency increases, the entropy in higher scales also increases. Furthermore, Figure 7d,f,h,j. demonstrate that all methods exhibit domain variations in the modulated signal. However, in scales smaller than 10 and window sizes ranging from 1 to 150, while both RCMFDE and MFDE are capable of indicating domain changes, RCMDE and MDE are less effective at detecting these domain variations within this segment of the signal and for scales below 10.

### 4.4. Sensitivity to Signal Length

In this section, we conduct a comparative analysis of RCMFDE, MFDE, RCMDE, and MDE in terms of their sensitivity to signal length. To achieve this objective, we employ WGN and 1f noise with varying sample points denoted as N. The signal lengths are systematically varied, spanning from 100 to 5000 samples. For each unique value of N, 100 independent WGN and 1f noise signals are generated.

**Figure 8:**
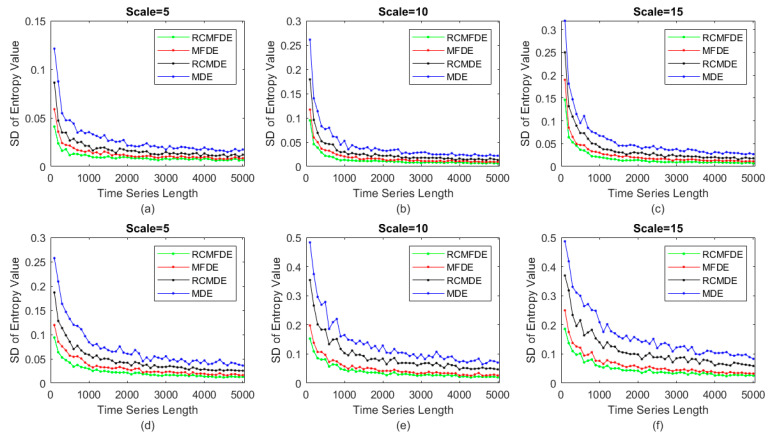
SD of results obtained from RCMDFE, MFDE, RCMDE, and MDE for 100 independent instances of 1f noise at scales (**a**) τ = 5, (**b**) τ = 10, and (**c**) τ = 15 and 100 independent instances of WGN at scales (**d**) τ = 5, (**e**) τ = 10, and (**f**) τ = 15.

The standard deviation (SD) of the obtained results at scale τ = 5, 10, and 15 is computed and presented in Figure 8. The findings underscore several key observations: Firstly, as the values of N increase, the corresponding SDs decrease, yielding more stable outcomes. Secondly, when comparing the SD of outcomes obtained via RCMFDE and MFDE with those of RCMDE and MDE, it becomes evident that the former exhibits lower SDs. Consequently, the outcomes obtained from RCMFDE and MFDE demonstrate greater stability compared to those of MDE and RCMDE.

### 4.5. Computation Time

**Figure 9:**
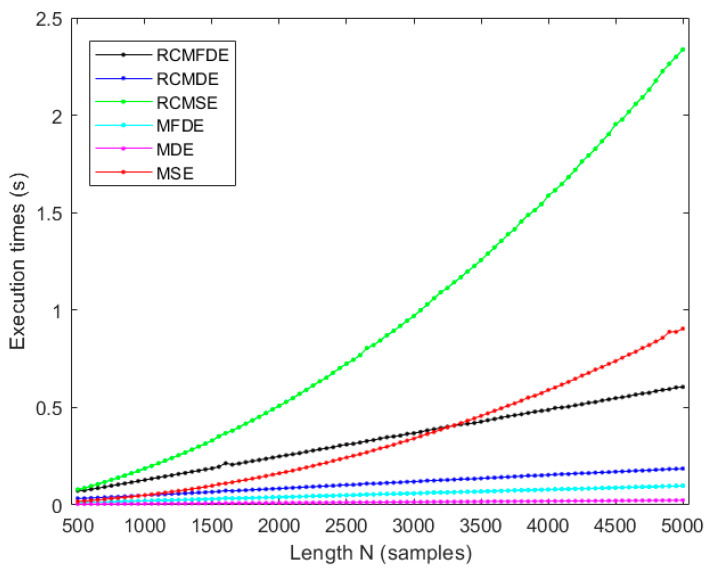
Evaluating the computational times of RCMFDE, RCMDE, RCMSE, MFDE, MDE, and RCMSE for white Gaussian noise (WGN) series of varying lengths.

To assess the computational efficiency of RCMFDE and RMFDE in comparison to RCMSE, MSE, RCMDE, and MDE, we employ the white Gaussian noise (WGN) sequences of varying lengths, ranging from 500 to 5000 sample points. The outcomes of these evaluations are illustrated in Figure 9. The simulations were executed on an Asus laptop equipped with an Intel(R) Core(TM) i5-8250U processor operating at 1.6 GHz and 8 GB of RAM, utilizing MATLAB R2021a.

As illustrated in Figure 9, although the computation time for RCMFDE is greater than that of RCMDE, and similarly, the computation time for MFDE exceeds that of MDE, the computation time for RCMFDE in comparison to RCMSE, as well as for RMFDE in comparison to RMSE, is significantly lower. These results are in agreement with the fact that the computational complexity of calculating SE is *O*(*N*^2^), while DE approaches have the computational complexity of *O*(*N*^2^) [18,72].

### 4.6. Simulated Bearing Signal Analysis

**Figure 10:**
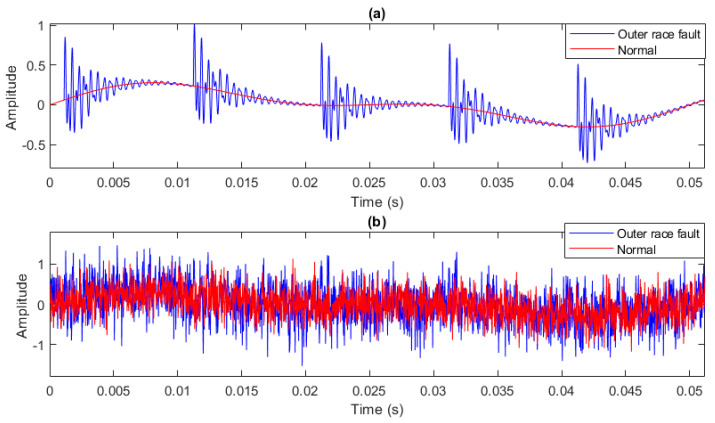
A simulated bearing signal: (**a**) without noise and (**b**) with noise.

According to Section 3.1.3, fifty independent signals of faulty and healthy bearings with a length of 2048 data points and a sampling frequency of 40 kHz are simulated in this section. Also, n(t) is assumed to be a WGN so that the variance of the signal-to-noise ratio (SNR) is 0.257 [73]. By eliminating the fault impulses, the healthy bearing signal is modeled. Figure 10 depicts an instance of simulated signals with and without noise.

**Figure 11:**
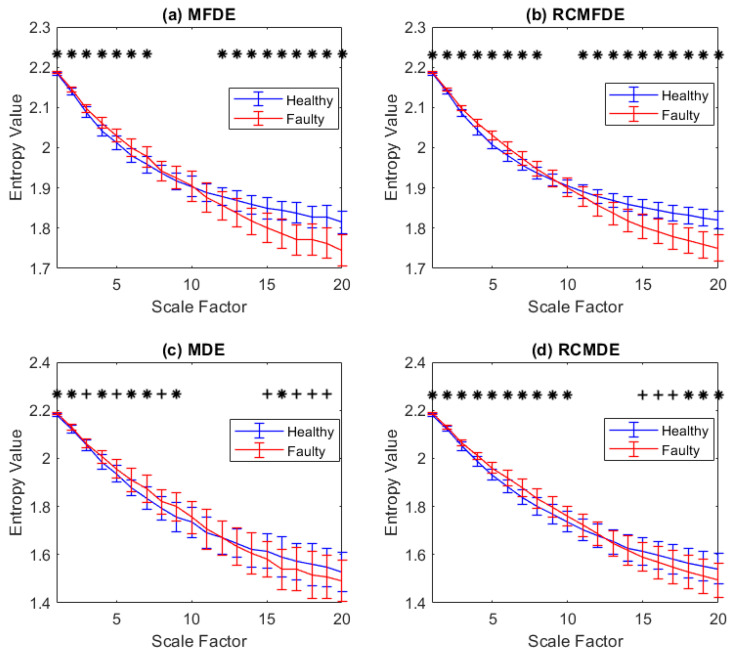
Mean value and SD of results of (**a**) MFDE, (**b**) RCMFDE, (**c**) MDE, and (**d**) RCMDE computed from healthy and faulty bearing simulated signals. The scale factors with *p*-values between 0.01 and 0.05, and smaller than 0.01 are respectively shown with + and *.

The MFDE, RCMFDE, MDE, and RCMDE of the simulated signals are calculated at 20 time scales. Based on the means and SDs of the results, as depicted in Figure 11, only the results of RCMFDE at three specific scales are distinctly separable without overlap, while the results of other methods exhibit overlap across all scales.

For each scale factor, Student’s *t*-test is used to examine statistical differences. The scale factors with a *p*-value between 0.05 and 0.01 (significant) and lower than 0.01 (very significant) are indicated with symbols + and ∗ in Figure 11. The RCMDE-based results have very significant difference at 18 scale factors (all scales except scales 9 and 10). However, MFDE, RCMDE, and MDE, respectively, lead to (very) significant differences at only 16, 16, and 14 scale factors. This fact suggests that RCMFDE has a higher capability of discrimination between the simulated signals of faulty and healthy bearings than MFDE, RCMDE, and MDE.

Furthermore, the Hedges’ g effect size [74] was employed to assess the distinguishing capability between simulated signals from faulty and healthy bearings. The results are presented in Table 4. The effect sizes of the RCMFDE and MFDE outcomes, when compared to RCMDE and MDE, consistently exhibit higher values across nearly all scales, except for three specific scales. This observation underscores the superior ability of RCMFDE and MFDE in distinguishing between simulated faulty bearings and healthy bearings. Moreover, the effect size of RCMFDE results consistently exceeds that of MFDE across all scales. Consequently, RCMFDE exhibits a greater capacity, relative to MFDE, in effectively distinguishing between simulated faulty bearings and healthy ones.

### 4.7. Noise Effect

In order to indicate the effect of adding noise to bearing signals, 50 independent realizations of WGNs were added to faulty bearing signals at different SNRs, and the sensitivities of MFDE, RCMFDE, MDE, and RCMDE to noise are evaluated. According to Section 3.1.3, fifty faulty bearing signals of 2048 data point length and a sampling frequency of 40 kHz are simulated without adding noise.

NrmEntN(*i*) is the measure of sensitivity to WGN in scale *i* [18].

(18) $$\mathrm{NrmEntN}(i)=\frac{\mathrm{entropy}\;\mathrm{of}\;a\;\mathrm{series}\;\mathrm{with}\;\mathrm{noise}\;\mathrm{in}\;\mathrm{scale}i}{\mathrm{entropy}\;\mathrm{of}\;a\;\mathrm{series}\;\mathrm{without}\;\mathrm{noise}\;\mathrm{in}\;\mathrm{scale}i}$$

**Figure 12:**
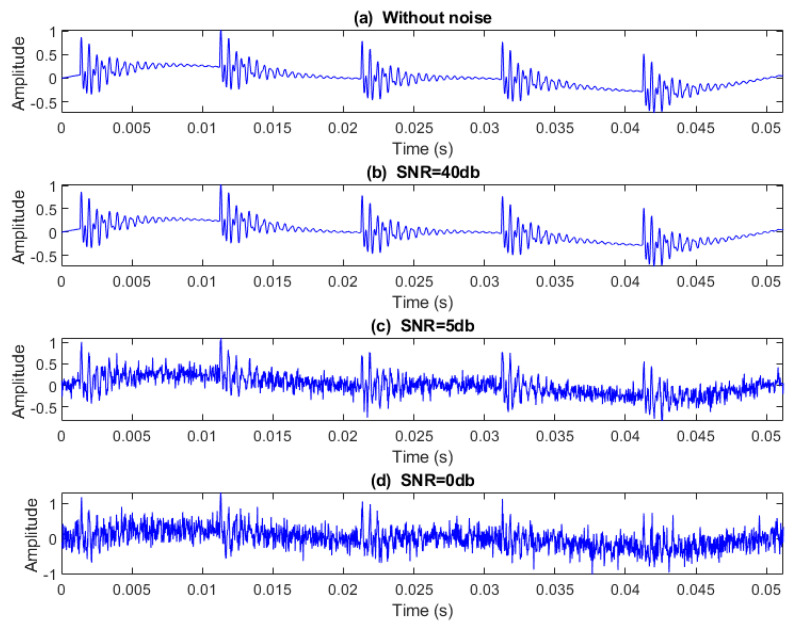
A simulated signal: (**a**) without noise and with additive WGN with respect to (**b**) SNR = 40 dB, (**c**) SNR = 5 dB, and (**d**) SNR = 0 dB.

**Figure 13:**
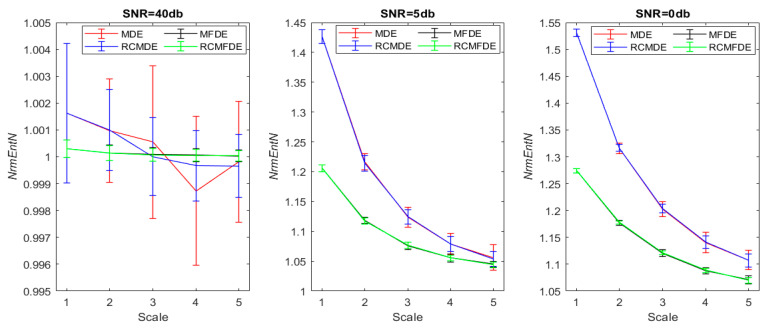
Average and SD of NrmEntN obtained via MDE, RCMDE, MFDE, and RCMFDE from 50 simulated faulty bearing signals with 50 independent additive realizations of WGNs relative to different noise power. NrmEntN compares the sensitivity of MDE, RCMDE, MFDE, and RCMFDE to WGN with different SNRs.

NrmEntN is calculated for MFDE, RCMFDE, MDE, and RCMDE for five scales by adding the WGN of different SNRs (0, 5, and 40 dB) to simulated signals. Figure 12 shows a simulated signal with/without the noise of different SNRs. Figure 13 and Table 5 present the average and SD of NrmEntN for different entropy methods over five scales.

The NrmEntN values obtained based on MFDE and RCMFDE, compared with MDE and RCMDE, have average values closer to 1 and also have a lower SD values. Therefore, MFDE and RCMFDE have lower sensitivities relative to WGN than MDE and RCMDE, and they are more resistant to noise. In addition, the SD of NrmEntN values in the RCMFDE method is lower than that for MFDE, indicating that RCMFDE is less sensitive to noise than MFDE.

### 4.8. Experimental Data Analysis

#### 4.8.1. Fault Diagnosis with respect to the Paderborn University Bearing Dataset

There are 60 measured datasets for each fault condition of the bearings. Five samples with a length of 2048 were separated from each measured dataset, thus generating 300 samples for each fault condition. MSE, RCMSE, MDE, RCMDE, MFDE, and RCMFDE were calculated for all signals over 20 scales. The results of each method were classified. From the signals for each fault condition, 480, 120, and 600 samples are utilized as training, validation, and test data, respectively.

A multiclass adaptive neuro-fuzzy inference system with fuzzy c-means clustering (FCM-ANFIS) [8] was used as the classifier in this study. A binary vector was applied as the target vector for each fault condition. Since four fault conditions were examined in this section, the length of each binary vector was 4, and the applied classifier was composed of four FCM-ANFIS; each determines one entry of the target vector.

**Figure 14:**
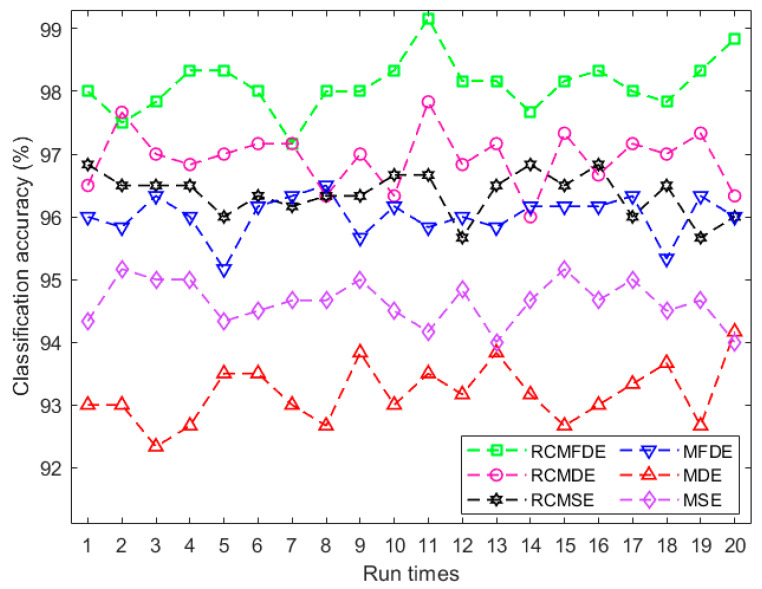
Classification of bearing fault conditions for MDE, RCMDE, MFDE, and RCMFDE using multiclass FCM-ANFIS.

The classification approach was repeated twenty times. The results are presented in Figure 14 and Table 6, indicating the higher average classification accuracy of features resulting from RCMFDE and MFDE compared to RCMDE and MDE, respectively. This suggests that MFDE and RCMFDE are more appropriate than MDE and RCMDE for pattern detection in bearing fault conditions. The highest average classification accuracy is 98.11%, obtained for features extracted by RCMFDE. This fact indicates that RCMFDE is the most suitable feature extraction method. Details of fault classification with the highest accuracy conducted by RCMFDE are presented in Table 7.

#### 4.8.2. Fault Diagnosis on PHMAP 2021 Data Challenge Dataset

In this section, we utilized three fault conditions from the PHMAP 2021 Data Challenge Dataset, as outlined in Section 2.2. For each of these conditions, we extracted three hundred independent signal samples, each consisting of 1024 data points.

We employed various multiscale entropy-based techniques, specifically MSE (m = 2, r = 0.15 × SD of original signal), RCMSE (m = 2, r = 0.15 × SD of original signal), MDE, RCMDE, MFDE, and RCMFDE, to analyze all signals across 20 different scales. The resulting values from these methods were employed as features for the purpose of fault diagnosis.

For the classification process, we utilized the multiclass FCM-ANFIS classifier [31]. Since we examined three distinct fault conditions, the target binary vector length was set to 3. The classifier was constructed using three FCM-ANFIS models. For each specific condition, we allocated 120 samples for training, 30 samples for validation, and 150 samples for testing. These data were classified 20 times using multiclass FCM-ANFIS.

**Figure 15:**
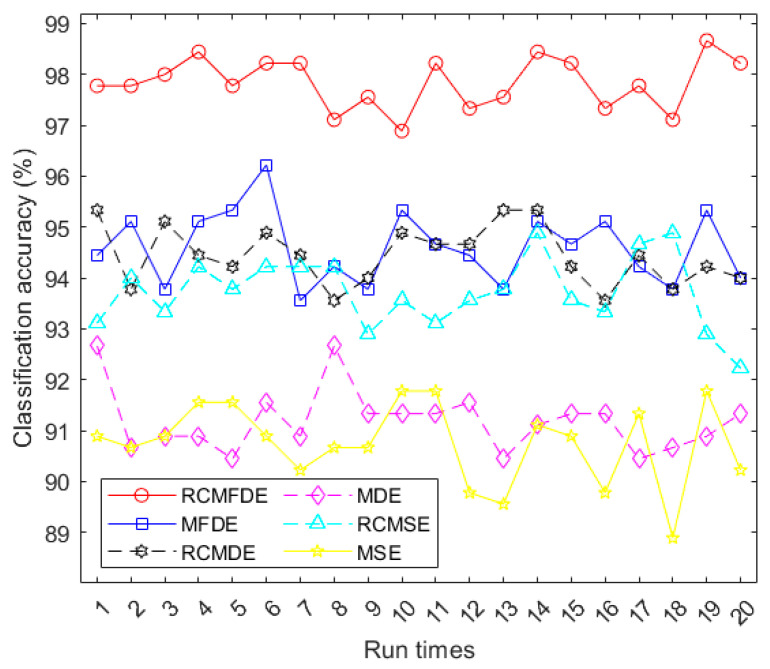
Accuracies of classifying the three fault conditions using multiclass FCM-ANFIS with different inputs.

The accuracies of this classification process are visualized in Figure 15 and summarized in Table 8. The results demonstrate the superior performance of RCMFDE when compared to other multiscale entropy algorithms in extracting relevant bearing features. Detailed information about the fault classification with the highest accuracy achieved using RCMFDE is presented in Table 9.

#### 4.8.3. Fault Diagnosis on the Case Western Reserve University (CWRU) Bearing Dataset

All 16 fault conditions from the CWRU bearing dataset were used when the sampling frequency was 12,000 Hz. The examined fault conditions are demonstrated in Table 10. For each of these conditions, we extracted 220 independent signal samples, each consisting of 2048 data points. In each condition, the motor shaft rotated at 1730, 1750, 1772, and 1797 rpm speeds.

Multiclass FCM-ANFIS was also used as the classifier for this dataset. Since 16 fault conditions were examined in this section, the target binary vector length was assumed to be 16, and the classifier is made from 16 FCM-ANFIS. The training dataset consisted of 80 signals, the validation dataset consisted of 20 signals, and the testing dataset consisted of 120 signals for each bearing fault condition.

MSE (*m* = 2, *r* = 0.15×SD of original signal), RCMSE (*m* = 2, *r* = 0.15×SD of original signal), MDE, RCMDE, MFDE, and RCMFDE were calculated for all signals, and the values of these methods were employed as features for the fault diagnosis.

**Figure 16:**
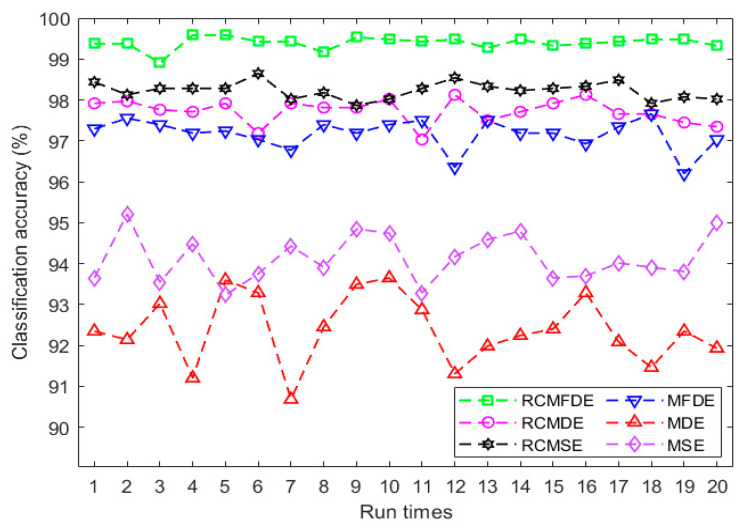
The classification results of bearing fault diagnosis using multiclass FCM-ANFIS with various inputs.

These data were classified by multiclass FCM-ANFIS. The results are presented in Figure 16 and Table 11, indicating the higher average classification accuracy based on RCMFDE. It suggests that RCMFDE is more appropriate than the other existing multiscale entropy algorithms for extracting bearing features. The details of fault classification with the highest accuracy using RCMFDE are presented in Table 12.

Considering the satisfactory results achieved with real and synthetic signals, the proposed method can find practical industrial applications. However, in future research endeavors, RCMFDE can be employed to utilize the processed data via other signal processing methods, such as wavelet, VMD, etc., further enhancing its fault detection capabilities in industrial applications.

## 5. Conclusions

This paper introduced RCMFDE as a measure of signal complexity and recommended using it when extracting the features of bearing vibration signals. RCMFDE, compared with MDE, MFDE, and RCMDE, calculated signal complexity with more reliability and stability, which was confirmed using different synthetic and real datasets. Although the behavior of (RC)MFDE was similar to (RC)MDE for white and pink noise, the former led to lower SDs and consequently was more stable than the latter. In simulated bearing signals, the results of RCMFDE indicated a significant difference between faulty and healthy conditions over the majority of scales. Additionally, (RC)MFDE resulted in higher resistance against noise than (RC)MDE. In fault diagnosis by three empirical datasets, features obtained from RCMFDE resulted in higher classification accuracy than MSE, RCMSE, MDE, RCMDE, and MFDE. Overall, these findings suggest the superiority of RCMFDE over the conventional multiscale entropy methods in bearing feature extraction.

## Figures and Tables

**Table 1:** Advantages, disadvantages and some applications of the most popular conventional entropy methods (SE, FE, PE and DE).

Methods	Advantages	Disadvantages	Some of Applications
SE	SE deals with the self-matching problem of approximate entropy and eliminates the bias in approximate entropy algorithm [17].	(1) SE may result in undefined or unreliable entropy values, especially for short time series [18]; (2) SE has high computational cost [19].	Mechanical [20], biomedical [21], civil engineering [22]
FE	FE, compared with SE, leads to more stable and accurate results [23].	(1) FE may result in undefined or unreliable entropy values, especially for short time series [19]. (2) Computational cost of FE is higher than SE [19].	Mechanical [24], biomedical [25],
PE	(1) PE is faster than SE and FE [16,26]; (2) PE value is more reliable than that for SE or FE for short signals [19].	(1) PE only captures order relations between amplitude values and ignores some signal information [18,27]; (2) PE neglects equal values in a signal; (3) PE is sensitive to a high SNR noise [18,27].	Mechanical [28], biomedical [29], economy [30], geophysics [31], hydrology [32]
DE	(1) Unlike SE, DE does not lead to undefined results in short signals [33]; (2) DE is less susceptible to the effects of noise [7]; (3) unlike PE, DE considers amplitude values [18]; (4) DE addresses the issue of equal adjacent amplitude values in PE [16]; (5) compared to SE and FE, DE is considerably faster [1,7].	DE is sensitive to its parameters, particularly the number of classes and embedding dimension [19].	Mechanical [7], biomedical [34], economy [35]

**Table 2:** Applied datasets for four different bearing fault conditions.

		Condition of Bearing		
	H	STO	DO	PO
Bearing Code	K001	KA01	KA06	KA07

**Table 3:** Operational conditions.

No.	Rotational Speed (rpm)	Load Torque (Nm)	Radial Force (N)
1	1500	0.7	1000
2	1500	0.1	1000
3	1500	0.7	400

**Table 4:** Differences in results for faulty bearing vs. healthy bearing obtained by RCMFDE, MFDE, RCMDE, and MDE based on Hedges’ *g* effect size.

Feature Extractor	Scale																			
	1	2	3	4	5	6	7	8	9	10	11	12	13	14	15	16	17	18	19	20
RCMFDE	1.21	1.19	1.65	1.96	1.85	1.48	1.06	0.59	0.18	0.19	0.58	0.97	1.36	1.67	1.89	2.05	2.17	2.28	2.37	2.43
MFDE	1.21	0.82	0.78	1.41	1.09	0.95	0.81	0.21	0.36	0.01	0.41	0.74	0.96	1.41	1.46	1.85	2.04	1.66	1.96	2.05
RCMDE	1.06	0.65	1.00	1.34	1.24	1.08	1.06	0.80	0.67	0.58	0.39	0.16	0.11	0.17	0.37	0.46	0.48	0.56	0.64	0.69
MDE	1.06	0.58	0.36	0.77	0.52	0.77	0.74	0.53	0.72	0.29	0.21	0.01	0.18	0.17	0.483	0.61	0.37	0.45	0.46	0.45

**Table 5:** Average and SD of NrmEntN obtained via MDE, RCMDE, MFDE, and RCMFDE from 50 simulated faulty bearing signals.

WGN	Method		Scale				
			1	2	3	4	5
SNR = 40 db	MDE	mean	1.0016	1.0010	1.0005	0.9987	0.9998
		SD	0.0026	0.0019	0.0028	0.0028	0.0023
	RCMDE	mean	1.0016	1.0010	1.0000	0.9997	0.9997
		SD	0.0026	0.0015	0.0015	0.0013	0.0012
	MFDE	mean	1.0003	1.0001	1.0001	1.0001	1.0000
		SD	3.2605 × 10^−4^	2.9003 × 10^−4^	2.5598 × 10^−4^	2.4340 × 10^−4^	2.1888 × 10^−4^
	RCMFDE	mean	1.0003	1.0001	1.0001	1.0001	1.0000
		SD	3.2605 × 10^−4^	2.7657 × 10^−4^	2.3455 × 10^−4^	2.1962 × 10^−4^	1.9927 × 10^−4^
SNR = 5 db	MDE	mean	1.4262	1.2168	1.1234	1.0792	1.056 0
		SD	0.0115	0.0138	0.0166	0.0179	0.0215
	RCMDE	mean	1.4262	1.2142	1.1243	1.079 0	1.0535
		SD	0.0115	0.0127	0.0122	0.0125	0.0125
	MFDE	mean	1.2057	1.1186	1.0756	1.0557	1.0452
		SD	0.0055	0.0056	0.0061	0.0067	0.0048
	RCMFDE	mean	1.2057	1.1176	1.0767	1.0557	1.0441
		SD	0.0055	0.0053	0.0052	0.0049	0.0044
SNR = 0 db	MDE	mean	1.5310	1.3157	1.2028	1.1404	1.1078
		SD	0.0067	0.0093	0.0142	0.0191	0.0183
	RCMDE	mean	1.5310	1.3165	1.2038	1.1412	1.1069
		SD	0.0067	0.0064	0.0081	0.0118	0.0122
	MFDE	mean	1.2741	1.1767	1.1207	1.0877	1.0709
		SD	0.0038	0.0046	0.0059	0.0059	0.0075
	RCMFDE	mean	1.2741	1.1777	1.1212	1.0883	1.0698
		SD	0.0038	0.0030	0.0035	0.0042	0.0048

**Table 6:** Classification results of bearing fault conditions: H, STO, DO, and PO conditions.

Feature Extractor	Accuracy (%)		
	Min	Mean	Max
RCMFDE	97.17	98.11	99.17
RCMDE	96.00	96.93	97.83
RCMSE	95.67	96.37	96.83
MFDE	95.17	96.02	96.50
MDE	92.33	93.18	94.17
MSE	94.00	94.64	95.17

**Table 7:** Confusion matrix of results with the highest classification accuracy using RCMFDE.

		True Condition				
		H	STO	DO	PO	Sensitivity (%)
Predicted condition	H	150	0	1	0	99.34
	STO	0	150	0	0	100
	DO	0	0	147	2	98.66
	PO	0	0	2	148	98.67
	Precision (%)	100	100	98	98.67	A * = 99.17

* A is accuracy.

**Table 8:** Classification results for different fault conditions (high looseness of the V-belt, faulty bearing, and fault-free condition) using multiclass FCM-ANFIS with different inputs.

Features	Accuracy (%)		
	Min	Mean	Max
RCMFDE	96.89	97.83	98.67
MFDE	93.56	94.60	96.22
RCMDE	93.56	94.444	95.33
MDE	90.44	91.19	92.67
RCMSE	92.22	93.72	94.89
MSE	88.89	90.74	91.78

**Table 9:** Confusion matrix of results with the highest classification accuracy using RCMFDE.

		True Condition			
		Belt Looseness High	Bearing Fault	Normal	Sensitivity (%)
Predicted condition	Belt Looseness High	147	0	3	98
	Bearing fault	2	150	0	98.68
	Normal	1	0	147	99.32
	Precision (%)	98	100	98	AC * = 98.67

* AC is the accuracy.

**Table 10:** Description of the bearing dataset.

Bearing Condition	Fault Diameter (mm)	Fault Position Relative to Load Zone	Label of Classification
Normal	0	-	1
Rolling element	0.1778	-	2
Rolling element	0.3556	-	3
Rolling element	0.5334	-	4
Rolling element	0.7112	-	5
Inner race	0.1778	-	6
Inner race	0.3556	-	7
Inner race	0.5334	-	8
Inner race	0.7112	-	9
Outer race	0.1778	Centered @ 6:00	10
Outer race	0.3556	Centered @ 6:00	11
Outer race	0.5334	Centered @ 6:00	12
Outer race	0.1778	Orthogonal @ 3:00	13
Outer race	0.5334	Orthogonal @ 3:00	14
Outer race	0.1778	Opposite @ 12:00	15
Outer race	0.5334	Opposite @ 12:00	16

**Table 11:** Classification of different bearing fault conditions.

Features	Accuracy (%)		
	Min	Mean	Max
RCMFDE	98.91	99.39	99.58
RCMDE	97.03	97.73	98.12
RCMSE	97.86	98.23	98.65
MFDE	96.20	97.17	97.66
MDE	90.68	92.38	93.65
MSE	93.23	94.13	95.21

**Table 12:** Confusion matrix of results with the highest classification accuracy using RCMFDE.

		True Condition																
	Class	1	2	3	4	5	6	7	8	9	10	11	12	13	14	15	16	Sensitivity
Predicted condition	1	120	0	0	0	0	0	0	0	0	0	0	0	0	0	0	0	100
	2	0	120	0	2	0	0	0	0	0	0	0	0	0	0	0	0	98.36
	3	0	0	118	0	0	0	0	0	0	0	0	0	0	0	0	0	100
	4	0	0	2	115	0	0	0	0	0	0	0	0	0	0	0	0	98.29
	5	0	0	0	0	120	0	0	0	0	0	0	0	0	0	0	0	100
	6	0	0	0	0	0	120	0	0	0	0	1	0	0	0	0	0	99.17
	7	0	0	0	1	0	0	120	0	0	0	0	0	0	0	0	0	99.17
	8	0	0	0	0	0	0	0	120	0	0	0	0	0	0	0	0	100
	9	0	0	0	0	0	0	0	0	120	0	0	0	0	0	0	0	100
	10	0	0	0	0	0	0	0	0	0	120	0	0	0	0	0	0	100
	11	0	0	0	0	0	0	0	0	0	0	119	0	0	0	0	0	100
	12	0	0	0	0	0	0	0	0	0	0	0	120	0	0	0	0	100
	13	0	0	0	0	0	0	0	0	0	0	0	0	120	0	0	0	100
	14	0	0	0	0	0	0	0	0	0	0	0	0	0	120	0	0	100
	15	0	0	0	2	0	0	0	0	0	0	0	0	0	0	120	0	98.36
	16	0	0	0	0	0	0	0	0	0	0	0	0	0	0	0	120	100
	Precision	100	100	98.33	95.83	100	100	100	100	100	100	99.17	100	100	100	100	100	A* = 99.58

* A is accuracy.

## Data Availability

The data that use in this study are openly available in CWRU datasets at https://engineering.case.edu/bearingdatacenter, PHMAP 2021 datasets at http://phmap.org/data-challenge, and KAt datasets at https://mb.uni-paderborn.de/kat/forschung/datacenter/bearing-datacenter/.

## Abbreviations

CG	Coarse graining
CMSE	Composite multiscale sample entropy
CWRU	Case Western Reserve University
DE	Dispersion entropy
DO	Drilling on the outer ring
FDE	Fuzzy dispersion entropy
FCM-ANFIS	Adaptive neuro-fuzzy inference system with fuzzy c-means
FE	Fuzzy entropy
H	Healthy condition
M.	Multiscale
MDE	Multiscale dispersion entropy
MFDE	Multiscale fuzzy dispersion entropy
MPE	Multiscale permutation entropy
MSE	Multiscale sample entropy
PE	Permutation entropy
PHMAP 2021	Asia Pacific Conference of the Prognostics and Health Management Society 2021
PO	Pitting on the outer ring
RC	Refined composite
RCMDE	Refined composite multiscale dispersion entropy
RCMFDE	Refined composite multiscale fuzzy dispersion entropy
RCMFE	Refined composite multiscale fuzzy entropy
RCMPE	Refined composite multiscale permutation entropy
RCMSE	Refined composite multiscale sample entropy
SE	Sample entropy
SD	Standard deviation
SNR	Signal-to-noise ratio
STO	Sharp trench on the outer ring
VMD	Variational mode decomposition
WGN	White Gaussian noise
